# Venetoclax in Combination with Azacitidine for the Treatment of Newly Diagnosed Acute Myeloid Leukemia: A Canadian Cost–Utility Analysis

**DOI:** 10.3390/curroncol29100592

**Published:** 2022-10-08

**Authors:** Kimberly Guinan, Karine Mathurin, Yunghan Au, Andre C. Schuh, Cat N. Bui, Xinglei Chai, Jean Lachaine

**Affiliations:** 1 PeriPharm Inc., Montréal, QC H2Y 2H4, Canada; 2 Faculty of Pharmacy, University de Montreal, Montreal, QC H3T 1J4, Canada; 3 AbbVie Corporation, Saint Laurent, QC H4S 1Z1, Canada; 4 Cancer Clinical Research Unit, Princess Margaret Cancer Center, Toronto, ON M5G 2C1, Canada; 5 AbbVie Corporation, North Chicago, IL 60064, USA; 6 Analysis Group Inc., Boston, MA 02199, USA

**Keywords:** acute myeloid leukemia, azacitidine, cost-utility analysis, health economics, venetoclax

## Abstract

Treatment for acute myeloid leukemia (AML) typically involves intensive chemotherapy (IC); however, there is an unmet need for approximately 50% of AML patients who are deemed unfit or ineligible for IC. The purpose of this study was to evaluate, from a Canadian perspective, the economic impact of venetoclax in combination with azacitidine (Ven+Aza) for the treatment of patients with newly diagnosed AML who are 75 years or older or who have comorbidities that preclude using IC. A lifetime partitioned survival model was developed to assess the cost-effectiveness of Ven+Aza compared with Aza. Health states included event-free survival, progressive/relapsed disease, and death. Efficacy parameters were based on the VIALE-A trial. Analyses were conducted from Ministry of Health (MoH) and societal perspectives. Over a lifetime horizon, Ven+Aza was associated with a gain of 1.65 quality-adjusted life years (QALYs) compared with Aza. From an MoH perspective, Ven+Aza and Aza were associated with total costs of $204,305 and $82,333, respectively, resulting in an incremental cost–utility ratio of $73,841/QALY. Results were similar from a societal perspective. This economic evaluation demonstrates that, in comparison with Aza, Ven+Aza is a cost-effective strategy for the treatment of patients with newly diagnosed AML who are deemed unfit for IC.

## 1. Introduction

Acute myeloid leukemia (AML), which is characterized by an abnormal proliferation of immature myeloid cells with secondary hematopoietic insufficiency that infiltrate bone marrow, blood, and other tissues, is the most common form of acute leukemia in adults [1,2,3]. Worldwide, leukemia is one of the most common forms of cancer, with the eleventh highest incidence [4]. The most recent statistics from the Canadian Cancer Society reported that 1090 Canadians were newly diagnosed with AML in 2016 [5]. Previously thought to be an incurable disease, the prognosis of AML patients began to change with the introduction of new therapies starting in the 1960s; however, while these options have significantly impacted the lives of younger individuals, older patients are still at a disadvantage with limited treatment options [3,6]. Approximately 40% of AML patients under the age of 60 years, who receive intensive chemotherapy (IC) and are candidates for transplant, will be cured of their disease, whereas this estimate is between 5% and 15% for those who are older [3,6]. Additionally, the median survival of older AML patients ranges between 5 and 10 months with the current standard of care [3]. Given that AML already has a substantial impact on healthcare resource utilization, its increasing incidence in Canada should raise concerns [7]. New drugs that improve survival, response rates, duration of response, and quality of life (QoL), thereby reducing transfusions and hospitalizations, could substantially reduce the economic burden caused by AML [8,9].

The standard of care of AML treatment is intensive chemotherapy (IC) [10]. However, patients older than 65 (and particularly >75) years of age have a higher prevalence of unfavourable cytogenetics and more comorbidities, making them ineligible for IC due to the high toxicity of these regimens [11,12,13]. Indeed, estimates suggest that nearly 50% of AML patients may be ineligible or unsuitable candidates to receive standard IC during the initial induction phase [14,15,16,17]. Treatment alternatives for “unfit or ineligible” patients are limited to low-intensity treatment, best supportive care (BSC), or clinical trials with investigational drugs. Hypomethylating agents (HMAs), namely azacitidine (Aza) and decitabine, have become the standard of care for treating older or IC-ineligible AML patients [16,18,19]; however, Aza is approved for use in Canada only for AML patients with 20–30% blasts [18]. Low-dose cytarabine (LDAC) has been a primary option for older or IC-ineligible AML patients for many years; however, its place in AML therapy has shifted due to its relatively modest complete response (CR) and survival rates, the evolution of newer generation agents, and improved genetic profiling [16,19,20,21,22,23,24]. Given the limited treatment options and modest survival improvements of current therapies for IC-ineligible AML patients, there remains an unmet medical need for the development of more effective and safe therapies that can also improve patients’ QoL.

Venetoclax in combination with Aza (Ven+Aza) is indicated for the treatment of patients with newly diagnosed AML who are 75 years or older or who have comorbidities that preclude use of IC. The efficacy and safety of venetoclax in combination with HMAs have been assessed in three studies of patients with newly diagnosed AML who were ineligible for IC [25,26,27]. The VIALE-A study (NCT02993523) was a phase 3, randomized, double-blind, placebo-controlled, multicenter study comparing the efficacy and safety of Ven+Aza versus placebo (Pbo+Aza) among treatment-naïve patients with confirmed AML who were ineligible for IC due to medical comorbidities or age ≥ 75 years [26]. The results of this study concluded that the median overall survival (mOS: 14.7 months with Ven+Aza versus 9.6 months with Aza; hazard ratio [HR] for death: 0.66, 95% confidence interval [CI]: 0.52–0.85, *p* < 0.001), complete remission rate, transfusion-independence, duration of remission, and time-to-deterioration in quality of life were more favourable in patients randomized to Ven+Aza compared with Pbo+Aza. The safety profile of Ven+Aza was consistent with known side effect profiles of both agents, with adverse events (AEs) consistent with expectations for an older AML population. The most recently updated National Comprehensive Cancer Network (NCCN) guidelines recommend Ven+Aza as a category 1 (uniform NCCN consensus based on high-level evidence) preferred intervention for AML patients aged ≥60 years and ineligible for IC, with or without actionable mutations [28].

In order to inform Canadian healthcare decision makers, we conducted an analysis of the costs and effectiveness of treating newly diagnosed AML patients ineligible for IC with Ven+Aza compared with Aza alone, which is the standard of care in Canada.

## 2. Materials and Methods

### 2.1. Model Structure

**Figure 1:**
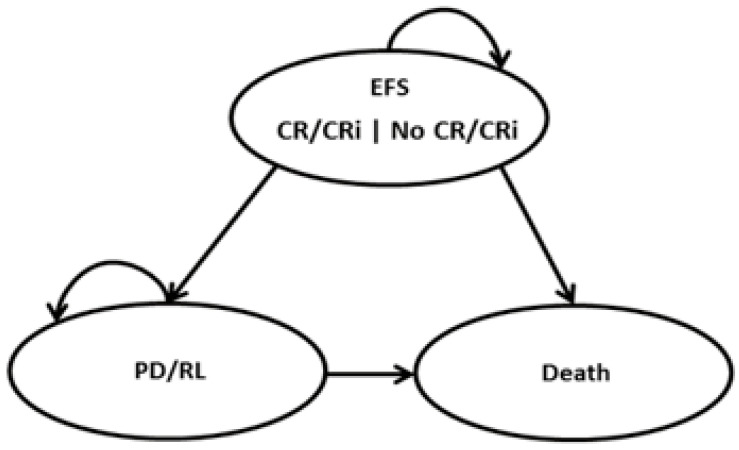
Partitioned survival model structure. EFS: Event-free survival, CR/CRi: complete remission/complete remission with incomplete blood count recovery, and PD/RL: progressive/relapsed disease.

A three-state partitioned survival model (PSM) was developed to assess, over a lifetime horizon, the economic impact of Ven+Aza versus Aza monotherapy and comprised three mutually exclusive health states: (i) event-free survival (EFS), (ii) progressive/relapsed disease (PD/RL), and (iii) death (Figure 1). EFS was defined as the time from the date of treatment initiation to the date of first documented progression or relapse from complete remission/complete remission with incomplete blood count recovery (CR/CRi), or treatment failure or death due to any cause. All patients began in EFS at the model start. The proportion of patients in the EFS health state of the model was set to be equal to the EFS curve of each treatment. Within EFS, a proportion of time was assumed in CR/CRi, which was estimated by applying the CR/CRi rate to the EFS curve. The remaining time was assumed not in CR/CRi. The PD/RL state included living patients who progressed or relapsed. The proportion of patients in the PD/RL health state was set to be equal to the difference between the proportion of living patients, which was based on the OS curve, and the proportion of EFS patients. During each cycle, patients were redistributed among the three health states, with death being the absorbing state. A 28-day model cycle was used for estimating the proportion of patients in each health state over time, according to the duration of treatment cycles.

### 2.2. Efficacy and Safety Data

Efficacy and safety inputs were extracted from the VIALE-A trial and included OS, EFS, and CR/CRi, which were derived from individual patient-level data (IPD) [26]. In the base-case analysis, the efficacy inputs for OS and EFS for Ven+Aza were predicted using parametric survival models. Fit of parametric survival models chosen were evaluated using several criteria, including the Akaike and Bayesian information criteria, visual inspection, examination of the log-cumulative hazard plots, testing the proportional hazard assumptions, clinical input, and external validation (Table 1) [29]. The parametric survival models were used to inform OS and EFS until year 5. Since Kaplan–Meier (KM) curves plateau at the end of the study period, especially in the Ven+Aza KM curves, a cure assumption was applied to long-term survivors in the EFS with CR/CRi health state, which was supported by clinical expert opinion. Specifically, it was deemed appropriate to consider “cured” patients who are still in the EFS with CR/CRi health state after 5 years, which was more conservative than other estimates noted in other cost-effectiveness AML models [30]. Afterwards, these patients were considered cured and were assumed to follow survival of the general population, which was modelled using the general population mortality based on the 2019 Canadian life tables [31]. Adverse events (AEs) with an incidence rate ≥ 5% in at least one treatment arm in the VIALE-A trial were considered [26].

### 2.3. Utilities

The pooled patient-level data of the EuroQol Group-5 Dimension-5 Level Instrument (EQ-5D-5L) from the VIALE-A and VIALE-C trials (venetoclax in combination with LDAC) were used to derive utilities as the populations studied in both trials were similar (AML patients aged 75 years or older or who have comorbidities that preclude use of IC) [36,37]. EQ-5D-5L scores were estimated based on individual dimension scores and adjusted using Canada preference-weights (Table 1) [32]. Disutility values associated with AEs were extracted from the literature [34]. AEs not reported in the literature were assumed to be equal to those under the same AE category or the average disutility of all AEs. Patients receiving subsequent hematopoietic stem cell transplantation (HSCT) were assumed to have additional HSCT disutility for a one-year period following HSCT [35]. The subsequent HSCT rates for Ven+Aza and Aza were obtained from the VIALE-A trial [26]. These estimates were validated from the expert opinion of a Canadian haemato-oncologist.

### 2.4. Cost Data

From a Canadian Ministry of Health (MoH) perspective, the model considered the following cost components: initial treatment costs (including drug and administration), subsequent HSCT costs, subsequent treatment costs (including drug and administration), AE costs associated with initial treatments, medical costs associated with health states (i.e., hospitalization, blood transfusion, and other monitoring costs), and terminal care costs. From a societal perspective, costs associated with productivity loss were also considered. These estimates were validated from the expert opinion of a Canadian haemato-oncologist. The resource use specific to Ven+Aza and Aza alone were obtained from the overall population in the VIALE-A trial [26]. The costs and resource use that were not available in the VIALE-A trial were obtained from various Canadian sources, including the literature, public databases, and a Canadian haemato-oncologist, to the extent feasible (Table 2). For detailed cost inputs, assumptions, and sources, refer to the Supplementary Materials. All costs estimated before 2020 were inflated to 2020 Canadian dollars using the Consumer Price Index (CPI) [38].

### 2.5. Incremental Cost–Utility Analysis

The effectiveness outcome was the average quality-adjusted life years (QALYs). The incremental QALYs were calculated as the difference in the average QALYs over the time horizon between the two treatments. The incremental cost–utility ratios (ICURs) were calculated by dividing the difference in total costs between Ven+Aza and Aza by the difference in QALYs between both treatment arms. Analyses were conducted from both a Canadian Ministry of Health (MoH) and a societal perspective. The cost-effectiveness of Ven+Aza versus Aza was assessed at a willingness-to-pay (WTP) threshold of $100,000/QALY.

### 2.6. Sensitivity Analyses

Both deterministic and probabilistic analyses were performed for this economic evaluation to assess the impact of the variation of each parameter on the base-case results. The robustness of the base-case results was assessed through deterministic sensitivity analyses (DSA). This was performed by varying each single variable individually within the lower and upper bounds of all key parameters. For this analysis, model parameters were varied using standard errors, standard deviations, or 95% confidence intervals (CIs), when available, and a range of ±25% for other parameters. A probabilistic sensitivity analysis (PSA) was also performed to assess the overall impact of uncertainty associated with study parameters. Simultaneous variations in all key parameters were performed using Monte Carlo simulations. A total of 5000 Monte Carlo simulations were performed using appropriate distributions (beta distribution bounded by 0 and 1 for probabilities and utility values, and gamma distribution for disutilities, treatment duration, and cost parameters). Results of the PSA were presented as cost-effectiveness acceptability curves and the probability of being cost-effective at a threshold of $100,000/QALY was estimated [57].

## 3. Results

### 3.1. Base-Case Analysis

Ven+Aza was associated with an average of 2.53 QALYs, compared with an average of 0.87 QALY for Aza, for a QALY gain of 1.65 (Table 3). From an MoH perspective, Ven+Aza and Aza were associated with total costs of $204,305 and $82,333 (difference of $121,973), respectively, which resulted in an ICUR of $73,841/QALY. From a societal perspective, Ven+Aza and Aza were associated with total costs of $177,510 and $77,955 (difference of $99,554), respectively. As a result, the ICUR of Ven+Aza versus Aza over a 5-year time horizon from this perspective was $60,269/QALY.

### 3.2. Sensitivity Analysis

**Figure 2:**
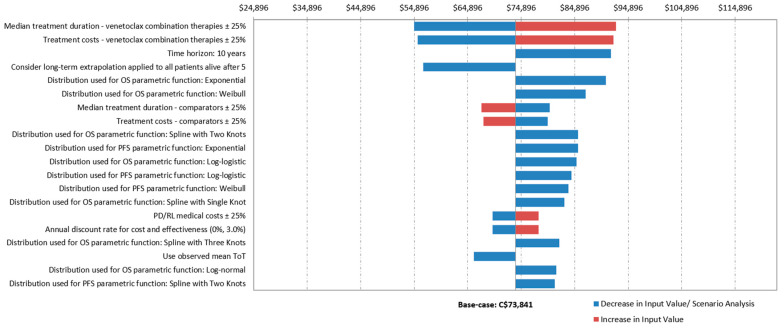
Results of one-way sensitivity analyses from a Ministry of Health perspective. $CA: Canadian dollars.

According to the DSA, the ICURs of Ven+Aza compared with Aza varied between $54,896/QALY and $92,659/QALY from an MoH perspective and between $41,324/QALY and $79,087/QALY from a societal perspective. The parameters with the greatest impact on the base-case ICURs from both MoH and societal perspectives were: (i) the median treatment duration of Ven+Aza, (ii) the treatment cost of Ven+Aza, (iii) the 10-year time horizon, and (iv) the consideration of long-term extrapolation applied to all patients alive after 5 years (Figure 2).

**Figure 3:**
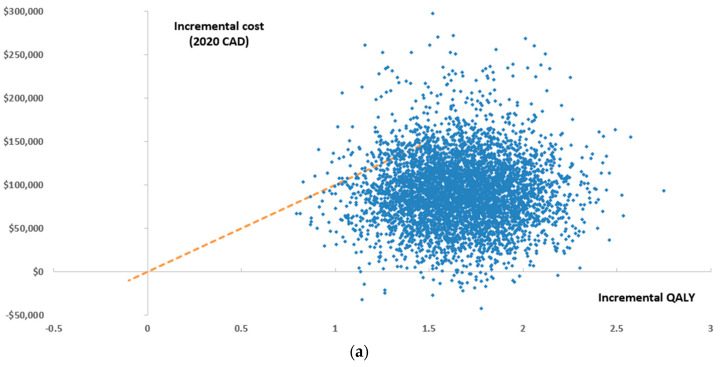

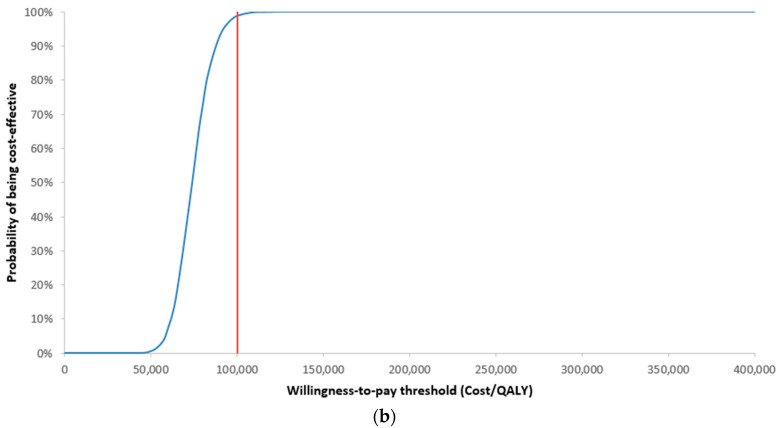
Results of the probabilistic sensitivity analysis from an MoH perspective presented as a (**a**) scatter plot and (**b**) cost-effectiveness acceptability curve, QALY: quality-adjusted life year.

According to a WTP threshold of $100,000/QALY, Ven+Aza was a cost-effective alternative compared with Aza in 98.8% of the Monte Carlo simulations from an MoH perspective (Figure 3a,b). From a societal perspective, Ven+Aza was cost-effective in 99.7% of Monte Carlo simulations.

## 4. Discussion

The objective of this study was to assess, from a Canadian perspective, the economic impact of Ven+Aza versus Aza alone for the treatment of patients with newly diagnosed AML who are 75 years or older or who have comorbidities that preclude the use of IC. Consequently, a PSM model was used, with health states including EFS, CR/CRi, and PD/RL, to model disease progression and evaluate treatment efficacy over the model time horizon. Efficacy data were obtained from the VIALE-A clinical trial. Utilities were obtained from clinical trials and the literature. Costs and resource use that were not available in the clinical trial were obtained from various Canadian sources, including the literature, public databases, and input of a Canadian clinical expert, to the extent feasible. Analyses were conducted from both a Canadian MoH and societal perspective by including costs associated with drug acquisition and administration, subsequent HSCT, subsequent treatments, medical costs, costs associated with AEs, follow-up, terminal care, and productivity loss. According to the probabilistic analysis from the MoH perspective, Ven+Aza is associated with an ICUR of $73,841/QALY compared with Aza.

A study was conducted from a US third-party payer perspective, using similar assumptions. The ICUR for VEN+AZA vs. AZA was estimated to be higher than the Canadian ICUR (US$96,579 per QALY gained), which may be due to the higher costs incurred in the US (drug acquisition costs, medical costs, etc.) compared with Canadian costs [58]. Another US study using a different approach also assessed the cost-effectiveness of Ven+Aza in unfit patients with previously untreated AML [59]. Results determined a QALY gain of 0.61 and incremental cost of $159,595, resulting in an ICUR of $260,343/QALY. However, this study differs in several respects from our Canadian analysis. First, US costs, including drug costs, are higher compared with Canada. Dose intensity was also not taken into consideration for drug costs calculations. Furthermore, our study was populated with individual patient-level data (IPD) as well as specific VIALE-A quality of life utility data (adjusted for Canada), which was not available in their study. In addition, our study also differentiated the higher utility value for patients in the EFS health state with CR/CRi, which was not considered in the US study. Based on these limitations, we contend that our Canadian analysis is more robust and reflective of the clinical reality.

As with any economic evaluation, there are inherent limitations to this cost–utility analysis. Although the lifetime horizon allowed the model to assess the cost-effectiveness of Ven+Aza over the lifespan of patients, several assumptions had to be made. Importantly, because the median follow-up in the VIALE-A trial was only 20.5 months for the data used in this analysis, it was necessary to project the KM curve for EFS and OS, as well as the median treatment duration, beyond the end of the follow-up period by fitting parametric survival distributions to the data from the trial; however, AML patients ineligible for IC are often older and have poor prognoses, with a 5-year OS rate of less than 10% [16]. Therefore, data extrapolation may not be aligned with the real-life survival of this patient population. These projections are associated with substantial uncertainty that may have a material impact on cost-effectiveness. In addition, the cure assumption included within the model is associated with some limitations. After year 5 of the model simulation, those who remained in the EFS with the CR/CRi health state were considered cured, though there is uncertainty around the treatment duration of patients in EFS with CR/CRi, specifically for those who are older and ineligible for IC. It is known that although some patients are in remission, treatment will be given continuously throughout the patient’s lifetime. However, in line with expert opinion, the cure assumption was still included within the model. That said, when “cure” is not considered, there is a difference of only $1696/QALY compared with the base-case analysis, demonstrating the minimal impact of this assumption on overall cost-effectiveness. Lastly, several inputs were based on clinical expert opinion, including the proportion of patients using subsequent treatments, the frequency of healthcare resource utilization per health state, as well as the proportion of AEs managed on an inpatient basis. The validation of the cure assumption within the model, established at 5 years, was also validated by clinical expert opinion. While management of the disease and health care resource utilization may vary across the country, these assumptions may have a material impact on the cost-effectiveness estimates.

## 5. Conclusions

AML is predominantly a disease of the elderly and is associated with poor survival. Patients older than 65 years of age have a higher prevalence of unfavourable cytogenetics and more comorbid medical conditions that make them ineligible for IC due to the high toxicity of these regimens. Indeed, less-fit, IC-ineligible AML patients who receive low-intensity treatment or BSC have an extremely poor prognosis, with a median 1-year survival rate of 15–20%. For IC-ineligible patients, achieving a CR or CRi is important but rarely achieved; this outcome provides longer survival and reduced symptom burden, including transfusion independence and improved QoL. Additionally, less intensive therapies for patients ineligible for IC do not provide durable responses or deeper remissions (approximately 18% achieve CR with HMAs or LDAC). Considering the favourable efficacy and safety profiles seen in the VIALE-A study and recommendations from recently updated key AML guidelines, Ven+Aza demonstrates a clear and indisputable therapeutic value. The current economic model predicted that Ven+Aza would offer marked benefits to patients with newly diagnosed AML who are ineligible for IC in terms of LYs and QALYs in comparison with Aza alone. Specifically, the results suggested that venetoclax was a highly effective treatment with good economic value. With an assumed per-cycle price of approximately $13,000 for Ven+Aza, the ICUR varied between $54,896/QALY and $92,659/QALY from an MoH perspective, which is quite acceptable for a safe and effective oncologic treatment that fulfils an unmet clinical need for patients with this fatal condition.

## Figures and Tables

**Table 1:** Model efficacy inputs.

Parameters	Model/Estimate	Reference
**Extrapolation Models**		VIALE-A trial [26]
OS—Ven+Aza	Log-normal	
OS—Aza	Exponential	
EFS—Ven+Aza	Gompertz	
EFS—Aza	Exponential	
**Utility inputs**		VIALE-A trial, VIALE-C trial, Xie (2016) [27,32,33]
EFS with CR/CRi	0.815	
EFS without CR/CRi	0.804	
PD/RL	0.733	
**Disutility Associated with AEs**		Wehler (2018) [34]
Hematological disorder	−0.090	
Atrial fibrillation	−0.121	
Dyspnea	−0.219	
Fatigue	−0.073	
Hemorrhage	−0.131	
Hypertension	−0.020	
Hypokalemia/Hypophosphataemia	−0.121	
Renal insufficiency	−0.218	
Sepsis/UTI/Pneumonia	−0.218	
**Subsequent HSCT**		
Rate—Ven+Aza	0.70%	Guadagnolo (2006) [35]
Rate—Aza	0.69%	
Disutility (Ven+Aza and Aza)	−0.0021	
Duration of disutility (cycles)	13	Assumption

AEs: Adverse events, Aza: azacitidine, CR/CRi: complete remission/complete remission with incomplete blood count recovery, EFS: event-free survival, HSCT: hematopoietic stem cell transplantation, OS: overall survival, PD/RL: progressive/relapsed disease, UTI: urinary tract infection, and Ven+Aza: venetoclax in combination with azacitidine.

**Table 2:** Model cost inputs (Canadian dollars).

Parameters	Cost/Estimate	Reference
**Drug and Administration Costs**		Drug cost: VIALE-A trial [26], IQVIA [39]; Administration costs: Government of Canada [40], Schedule of Benefits [41], CCO Regimens [42], Job Bank Canada [43], Pettigrew (2015) [44]
Ven+Aza		
First cycle	$10,526	
Subsequent cycles	$10,736	
Aza	$6355	
**Subsequent Treatment Cost by First-Line Treatment**		IQVIA [39], Dosing for Aza: VIALE-A [26], Dosing for cytarabine: VIALE-C [33], Dosing for hydroxycarbamide: CCO, HYDR regimen [45], Stahl (2018) [46]
Ven+Aza	$384.30	
Aza	$811.90	
**Subsequent HSCT**		OCC [47] Interprovincial Billing Rates for Designated High Cost Transplants, 2020 [48]
Stem cell harvesting cost	$2475	
Cost associated with allogeneic SCT procedure	$183,244	
**Medical Costs by Health State**		
EFS with CR/Cri		CIHI, Code 624 (adults) [49], CIHI 2016 [50], Lagerquist (2017) [51], OCC [47]
First Cycle	$7940	
Subsequent cycles	$668.76	
EFS without CR/Cri		
First Cycle	$8497	
Subsequent cycles	$1434	
PD/RL	$2654	
**Adverse Events by Treatment**		
Ven+Aza	$5699	VIALE-A trial [26], OCC 2017/2018 [47], OCC [47]
Aza	$3743	
**Follow-Up Costs by Health State**	$668.76	Canadian KOL input, Schedule of Benefits for Laboratory Services [52], Canadian Cancer Society [53]
**Costs of Terminal Care**	$86,582	de Oliveira (2016) [54]
**Costs of Productivity Loss**		
Average monthly wage	$4700	Statistics Canada. Table: 14-10-0320-02 [55]
Employment rate (%)		
15–24 years old	49.2	Statistics Canada. Table: 14-10-0287-02 [56]
25–55 years old	79.7	
55 years and older	33.7	

All costs were inflated to 2020 Canadian dollars using the CPI [38]. Aza: azacytidine, CIHI: Canadian Institute for Health Information, CR/Cri: complete remission/complete remission with incomplete blood count recovery, EFS: event-free survival, HSCT: hematopoietic stem cell transplantation, KOL: key opinion leader, OCC: Ontario Care Costing, PD/RL: progressive/relapsed disease, and Ven+Aza: venetoclax in combination with azacitidine.

**Table 3:** Cost–utility results.

	Aza	Ven+Aza
Total QALYs	0.87	2.53
Incremental QALYs		1.65
Total costs, MoH perspective	$82,333	$204,305
Incremental total costs, MoH perspective		$121,973
Total costs, Societal perspective	$77,955	$177,510
Incremental total costs, Societal perspective		$99,554
Incremental costs/QALY, MoH perspective		$73,841/QALY
Incremental costs/QALY, Societal perspective		$60,269/QALY

MoH: Ministry of Health, QALY: quality-adjusted life year.

## Data Availability

Data sharing is not applicable to this article.

## Supplementary Materials

The following supporting information can be downloaded at: https://www.mdpi.com/article/10.3390/curroncol29100592/s1, Table S1: Proportion of Patients Receiving Subsequent Treatments by First-Line Treatment; Table S2: Administration Cost Parameters for Oral Therapies; Table S3: Administration Cost Parameters for IV Therapies; Table S4: Administration Costs per Treatment; Table S5: Unit Costs of Hospitalization and Blood Transfusion; Table S6: Health Care Resource Utilization by Health State; Table S7: Grade 3/4 AEs ≥ 5% for Each Treatment; Table S8: Follow-up Costs; Table S9: Follow-up and Monitoring Frequency by Health State; Table S10: Follow-up and Monitoring Costs by Health State; Table S11: Costs of Terminal Care; Table S12: Indirect Cost Parameters; Table S13: Employment Rate. References [60,61,62] are citied in the Supplementary Materials.
