# Supplementary material for: Venetoclax in Combination with Azacitidine for the Treatment of Newly Diagnosed Acute Myeloid Leukemia: A Canadian Cost–Utility Analysis
Source: Curr Oncol. 2022 Oct 8;29(10):7524–36. doi: 10.3390/curroncol29100592 (PMC9601219; doi:10.3390/curroncol29100592)
Supplement: Supplementary file 1 [file curroncol-29-00592-s001.zip › curroncol-1860337-supplementary.pdf]

Current Oncology

MDPI

Venetoclax in Combination with Azacitidine for the Treatment of Newly Diagnosed Acute Myeloid Leukemia: A Canadian Cost–Utility Analysis

Supplementary Materials

Drug Acquisition and Administration Costs

Drug Acquisition Costs

- First-line treatments: the drug acquisition costs were calculated as a function of unit drug costs (IQVIA price list, October 2020) as well as vial sharing, dosing, relative dose intensity and treatment duration obtained from the VIALE-A.[1] Exponential model was used to extrapolate the time on treatment beyond the trial observation period.
- Subsequent treatments: The model assumed patients could also receive subsequent pharmacological treatments once they experienced PD/RL after the initial treatment, which reflected the natural treatment course patients experienced. Costs were applied to the proportion of patients who received subsequent treatments for each arm (≥5%), as observed in the clinical trials. Specifically, the proportion of patients receiving subsequent treatments for Ven+Aza and Aza arms were obtained from Canadian KOL input.
    - The dosing schedule for Aza was obtained from VIALE-A trial protocol,[1] from the VIALE-C trial for cytarabine[2], and from CCO HYDR regimen for hydroxycarbamide.[3] The unit drug costs were also obtained from IQVIA price list (October 2020).[4] The mean treatment duration of Aza as subsequent treatment was derived from Stahl (2018), which is a retrospective database study that evaluated HMAs, including Aza, in relapsed/refractory (RR) AML patients.[5] Treatment durations of other subsequent therapies were assumed to be the same as Aza. , and from CCO HYDR regimen for hydroxycarbamide.[3]

Table S1. Proportion of Patients Receiving Subsequent Treatments by First-Line Treatment.

**Subsequent Treatments**	**First-Line Treatments**	
	**Ven+Aza**	**Aza**
Aza	0.0%	0.0%
LDAC	5.0%	12.0%
Hydroxycarbamide	10.0%	10.0%
Source	Canadian KOL input	

Abbreviations: Aza: Azacitidine; **KOL**: Key opinion leader; **LDAC**: low-dose cytarabine; **Ven+Aza:** venetoclax in combination with azacitidine

Administration Costs

• Administration costs were informed by administrative information for chemotherapy regimens retrieved from Cancer Care Ontario (CCO).[6] Administration costs were informed by administrative information for chemotherapy regimens retrieved from Cancer Care Ontario (CCO).[6]

• The administration cost of oral treatments (venetoclax and hydroxcarbamide) was assumed to be associated with pharmacy dispensing fee as well as a physician fee for the management/supervision of oral chemotherapy. The dispensing fee in Ontario was selected,[7] and the physician fee was obtained from the Schedule of Benefits for Physician Services [8].

• For Aza, which is administered intravenously (IV), information regarding administration retrieved from Cancer Care Ontario (CCO) were used to estimate administration costs. IV administration cost was based on pharmacist, nurse, and physician workloads, as well as chair time (overhead costs). Nursing and pharmacy workloads were obtained from CCO and multiplied by their respective median wage, obtained from Job Bank Canada.[9] In addition, the physician cost was obtained from the Schedule of Benefits for Physician Services, by using a specific cost associated with chemotherapy administration.[8] Lastly, the cost of IV administration also included overhead costs. To estimate these costs, the mean duration of patient visit for each treatment regimen was also obtained from CCO and was multiplied by the average infusion cost per minute, obtained from Pettigrew (2016).[6,10]

Table S2 : Administration Cost Parameters for Oral Therapies

Resource	Cost	Sources
Dispensing fee (per claim)	$8.83	Government of Canada. Dispensing Fee Policies in Public Drug Plans, 2017/2018.[7]
Physician	$25.75	Schedule of Benefits. Code G388.[8]
**Total per cycle**	**$34.58**	

Table S3: Administration Cost Parameters for IV Therapies

Resource	Average Cost ($/min)	Sources
Pharmacist	$0.89	CCO Regimens [6]; Statistic Canada (Table: 14-10-0043-01)[11]; Job Bank Canada (NOC 3131- Canada).[9]CCO Regimens [6]; Statistic Canada (Table: 14-10-0043-01)[11]; Job Bank Canada (NOC 3131- Canada).[9]
Nurse	$0.63	CCO Regimens [6]; Job Bank Canada (NOC 3131- Canada).[9]
Physician	$75.00 (per visit)	Schedule of Benefits. Code G345[8]
Chair time	$0.77	Pettigrew (2015).[10]

Abbreviations: CCO: Cancer Care Ontario,

Table S4: Administration Costs per Treatment

Treatment Regimen	Route of administration	Pharmacy Time (min)	Nursing Time (min)	Patient Visit (min)	Weighted Average Cost per Visit	Cost per Cycle	Source
**Ven+Aza**							
Venetoclax	PO	-	-	-	-	$34.58	Oral admin
Aza	IV + PO	11.88	27.50	30	$50.91 (per admin)	$868.91	CCO, VENAZA Regimen.[12]

**Aza**	IV	11.88	27.50	30	$50.91 (per admin)	$868.91	CCO, VENAZA Regimen.[12]
**LDAC**	IV	18.29	76.67	0	$139.36 (per admin)	$1,393.60	CCO, CYTA(LD) Regimen.[13]
**Hydroxycarbamide**	PO	-	-	-	-	$34.58	Oral admin

Abbreviations: Aza: azacitidine; **CCO:** Cancer Care Ontario, **IV** intravenous; **LDAC:** low-dose cytarabine; **PO:** per os; **Ven+Aza**: venetoclax in combination with azacitidine

Subsequent Hematopoietic Stem Cell Transplantation Costs

- The model assumed patients could receive subsequent HSCT after initial treatment. Cost and disutility were added separately for the proportion of patients who received subsequent HSCT for each arm.
- HSCT costs were considered in two parts: stem cell harvesting costs and HSCT procedure/follow-up cost (assuming a length of stay of 25 days). The stem cell harvesting cost was retrieved from the Ontario Care Costing (OCC) Analysis Tool.[14] Only the allogeneic stem cell transplantation (SCT) was considered because it is the most common SCT performed in AML patients.[15] The costs associated with allogenic SCT procedure and follow-up were retrieved from the interprovincial billing rates for designated high cost transplants, using the most recent 2020 report.[16]

- HSCT costs were considered in two parts: stem cell harvesting costs and HSCT procedure/follow-up cost (assuming a length of stay of 25 days). The stem cell harvesting cost was retrieved from the Ontario Care Costing (OCC) Analysis Tool.[14] Only the allogeneic stem cell transplantation (SCT) was considered because it is the most common SCT performed in AML patients.[15] The costs associated with allogenic SCT procedure and follow-up were retrieved from the interprovincial billing rates for designated high cost transplants, using the most recent 2020 report.[16]

Medical Costs Associated with Health States

- The medical costs included hospitalization, blood transfusion, and other monitoring costs that consisted of physical check-ups and routine monitoring labs/procedures. During the ramp-up treatment initiation period, 100% patients who received Ven+Aza were hospitalized for 4 and 5 days, respectively, based on the VIALE-A trial protocol.[1] The same hospitalization rate and length of stay (LOS) were considered for the Aza arm. Afterwards, the hospitalization costs were assumed to be independent of treatment, instead, they would vary by health states and time horizon until year 5. The average LOS per cycle for each health state (i.e., EFS state with CR/CRi achieved, EFS state without CR/CRi achieved, PD/RL state) was derived based on Canadian KOL input. After year 5, patients were considered cured and it was assumed that they would not incur much resource use, and therefore would incur costs for AML long-term survivors. In the base-case model, the medical costs associated with long-term survivors were assumed to be equal to those in the "EFS with CR/CRi" state.
- Similarly, blood transfusion costs and other monitoring costs were assumed to be independent of treatments received and only depended on health states. The resource use of red and platelet blood transfusion per cycle for each health state were also derived from Canadian KOL input.

- A mean hospitalization cost per day for AML diagnosis was retrieved for all adult patients, from the Patient Cost Estimator provided by the Canadian Institute for Health information (CIHI).[17] The main daily cost in intensive care unit (ICU) was retrieved from the CIHI 2019 report, for care in Canadian ICUs.[18] For both ambulatory care of AML and platelet transfusion, costs were retrieved from the OCC Analysis Tool.[14] Finally, the cost of red blood cell transfusion was retrieved from the study by Lagerquist (2017).[19]

Table S5: Unit Costs of Hospitalization and Blood Transfusion

Service	Cost	Sources
Inpatient	$1,817.86	CIHI, Patient Cost Estimator 2018. Code 624 (adults).[17]
ICU	$3,927.67	CIHI 2016.[18]
Reb blood transfusion	$265.82	Lagerquist (2017) - red blood cell transfusion.[19]
Platelet transfusion	$476.17	OCC. Codes Code 1LZ19HHU4J. Ambulatory care, 2017/2018.[14]

Abbreviations: CIHI: Canadian Institute for Health Information; **ICU**: Intensive Care Unit; **OCC**: Ontario Case Costing

Table S6: Health Care Resource Utilization by Health State

Health state	Inpatient LOS per cycle	Number of red blood cell transfusion per cycle	Number of platelet transfusion per cycle	Source
EFS with CR/CRi	0.00	0.00	0.00	Canadian KOL input
EFS without CR/CRi	0.12	0.75	0.75	
PD/RL	0.15	2.30	2.30	

Abbreviations: CR: complete remission; **CRi:** complete remission with incomplete blood count recovery; **EFS:** event-free survival; **KOL:** Key opinion leader; **PD/RL:** progressive/relapsed disease

Costs Associated with Adverse Events

- AE costs were calculated for Ven+Aza and Aza, based on rates of AE extracted from the VIALE-A trial (grades 3 or 4 AEs with ≥ 5%) and unit costs per AE from OCC.[1],[14] The proportion of AEs managed inpatient or outpatient was established based Canadian KOL input.
- The cost per event for both outpatient and inpatient management was also obtained from the OCC, for all AEs.[14]

Table S7: Grade 3/4 AEs ≥ 5% for Each Treatment

Grade 3/4 AEs ≥ 5%	Ven+Aza	Aza	Unit Cost per AE*	OCC Code[14]
Anemia	26.1%	20.1%	$1,022.23	D649
Atrial fibrillation	5.7%	2.1%	$1,434.58	I4890
Febrile neutropenia	41.7%	18.8%	$8,521.10	R508
Hypertension	6.0%	4.2%	$625.75	R030
Hypokalemia	10.6%	10.4%	$1,476.86	E876
Hypophosphataemia	7.4%	7.6%	$447.44	E878
Leukopenia	20.5%	11.8%	$571.61	D728
Neutropenia	42.0%	28.5%	$521.33	D700
Pneumonia	17.7%	25.0%	$2,502.74	J189
Sepsis	5.7%	6.9%	$7,590.56	A419
Thrombocytopenia	44.5%	38.2%	$440.26	D696
Urinary tract infection	3.9%	5.6%	$729.35	N390
Source	VIALE-A trial[1]	VIALE-A trial[1]	-	-
**Total AE Cost**	**$5,698.60**	**$3,742.62**	-	-

Abbreviations: AE: Adverse event; **OCC**: Ontario Case Costing

Follow-up Costs

- According to the Canadian Cancer Society, follow-up and monitoring of AML include complete blood count (CBC), blood chemistry tests (liver, renal and electrolyte panels), imaging test (chest x-ray, computed tomography [CT] scan, magnetic resonance imaging [MRI] or ultrasound) and bone marrow aspiration and biopsy.[20] A follow-up visit with a hematologist was also included for routine care. [20]
- Monitoring costs were mostly obtained from the Schedule of Benefits for Physician Services and from the Schedule of Benefits for Laboratory Services.[21] The OCC Analysis Tool was also used to retrieve the procedure costs for bone marrow aspirates and biopsies.[14]

Table S8: Follow-up Costs

Description	Unit Cost	Source
Hematologist (consultation)	$168.75	Schedule of Benefits. Code A615.[8]
Hematologist (repeat consultation)	$105.25	Schedule of Benefits. Code A616.[8]
Bone marrow aspirates	$935.92	OCC. Codes 1WY58HA, 1WY58HN. Ambulatory care[14]
Bone marrow biopsies	$1,217.73	OCC. Code 2WY71HA[14]
Peripheral blood smears	$25.42	Schedule of Benefits for Laboratory Services, code L624 and Code L700.[21]
Blood tests	$16.06	Schedule of Benefits. Code G481.[8] Schedule of Benefits for Laboratory Services, code L393 and Code L700.[21]
Blood chemistry – liver panel	$21.15	Schedule of Benefits for Laboratory Services. Code L223, 222, 191,029,030, 031, 005, 208 and collection fee (code L700).[21]
Blood chemistry – renal panel	$13.33	Schedule of Benefits for Laboratory Services. Code L251,067 and collection fee (code L700).[21]
Blood chemistry – electrolyte panel	$18.08	Schedule of Benefits for Laboratory Services. Code L226, 204, 053,165,194,061 and collection fee (code L700).[21]
Chest X-ray	$32.60	Schedule of Benefits. Code X090[21]
CT-Scan	$108.30	Schedule of Benefits. Code X126 [21]

Abbreviations: CT: computed tomography; **MRI:** magnetic resonance imaging; **OCC**: Ontario Case Costing

Table S9: Follow-up and Monitoring Frequency by Health State

Health state	Frequency per Cycle			Source
	Remission	Stable Disease	Progressive Disease	
Hematologist	1	1	1	Canadian KOL input
Bone marrow aspirates	0.15	0.15	0.15	
Bone marrow biopsies	0.15	0.15	0.15	
Peripheral blood smears	4.00	4.00	4.00	
Blood tests	4.00	4.00	4.00	
Serum blood chemistry	4.00	4.00	4.00	
Chest X-ray	0.15	0.15	0.15	
CT-Scan	0.15	0.15	0.15	

Abbreviations: CT: computed tomography; **MRI:** magnetic resonance imaging

Table S10: Follow-up and Monitoring Costs by Health State

Health state	Other monitoring cost per cycle	Source
EFS with CR/CRi	$668.76	Canadian KOL input.
EFS without CR/CRi	$668.76	
PD/RL	$668.76	

Abbreviations: CR: complete remission; **CRi:** complete remission with incomplete blood count recovery; **EFS:** event-free survival; **PD/RL:** progressive/relapsed disease

Costs Associated with Long-Term Survivors

- Given the cure assumption applied for patients in the EFS with CR/Cri health state after 5 years, it was also assumed that these patients would not incur further costs associated with AML, and therefore, would have healthcare resource utilization similar to the general population. Consequently, no further costs were applied to those patients.

Costs Associated with Terminal Care

• All patients who transitioned to death were assumed to incur terminal care costs the last cycle before death.

• The cost of terminal care was obtained from a Canadian study by de Oliveira (2016).[22] A cost of terminal care for leukemia associated with the last 12 months of life was estimated at $72,194.[22]

Table S11: Costs of Terminal Care

	Cost per Event	Source
Male	$89,776.05	de Oliveira (2016)[22]
Female	$83,388.58	
**Average**	**$86,582.31**	

Costs Associated with Productivity Loss

- Costs pertaining to AML-related productivity loss were considered, from a societal perspective.
- In order to calculate the work productivity impact associated with AML, the average hourly wage, as well as the average weekly hours and employment rate were retrieved from Statistics Canada (**Table 2**).[11,23] This information was obtained for men and female, divided by three age groups: 15 to 24 years old, 25 to 54 years old and 55 years and older.

Table S12: Indirect Cost Parameters

Parameter	Value	Source
Average hourly wage* ($/h)	$31.67	Statistics Canada. Table: 14-10-0320-02.[11]<br>*Men and women, 25 years and over
Average number of hours per week* (h)	37.1	
Average monthly wage ($)	**$4,699.83**	

Table S13: Employment Rate

Age	Employment Rate (%)	Source
15-24 years old	49.2	Statistics Canada. Table 14-10-0287-02.[23]
25-55 years old	79.7	
55 years and older	33.7	

References

1. Dinardo, C.D.; Jonas, B.A.; Pullarkat, V.; Thirman, M.J.; Garcia, J.S.; Wei, A.H.; Konopleva, M.; Döhner, H.; Letai, A.; Fenaux, P.; et al. Azacitidine and Venetoclax in Previously Untreated Acute Myeloid Leukemia. *N. Engl. J. Med.* **2020**, *383*, 617–629, doi:10.1056/nejmoa2012971.
2. Wei, A.H.; Montesinos, P.; Ivanov, V.; Dinardo, C.D.; Novak, J.; Laribi, K.; Kim, I.; Stevens, D.A.; Fiedler, W.; Pagoni, M.; et al. Venetoclax plus LDAC for newly diagnosed AML ineligible for intensive chemotherapy: a phase 3 randomized placebo-controlled trial. *Blood* **2020**, *135*, 2137–2145, https://doi.org/10.1182/blood.2020004856.
3. Cancer Care Ontario. HYDR Regimen. Available online: https://www.cancercareontario.ca/en/drugformulary/regimens/monograph/51466 (accessed on 1 November 2020).
4. IQVIA. Delta PA. October 2020.
5. Stahl, M.; DeVeaux, M.; Montesinos, P.; Itzykson, R.; Ritchie, E.K.; Sekeres, M.A.; Barnard, J.D.; Podoltsev, N.A.; Brunner, A.M.; Komrokji, R.S. Hypomethylating agents in relapsed and refractory AML: Outcomes and their predictors in a large international patient cohort. *Blood Advances* **2018**, *2*, 923–932
6. Cancer Care Ontario. Drug Formulary - Regimens Available online: https://www.cancercareontario.ca/en/drugformulary/regimens?f%5B0%5D=field_type_of_cancer%3A626 (accessed on 1 October 2020).
7. Government of Canada. Dispensing Fee Policies in Public Drug Plans, 2017/18. Available online: http://www.pmprb-cepmb.gc.ca/view.asp?ccid=1308&lang=en (accessed on 1 October 2020).
8. Ministry of Health and Long Term Care of Ontario. Schedule of Benefits: Physician Services Under the Health Insurance Act (Effective April 1, 2020). Available online: http://www.health.gov.on.ca/en/pro/programs/ohip/sob/physserv/sob_master20200306.pdf (accessed on 1 October 2020).
9. Government of Canada. Job Bank - Wage Report. Available online: https://www.jobbank.gc.ca/wagereport/location/geo9219 (accessed on 1 October 2020).
10. Pettigrew, M.; Kavan, P.; Surprenant, L.; Lim, H. Comparative net cost impact of the utilization of panitumumab versus cetuximab for the treatment of patients with metastatic colorectal cancer in Canada. *Journal of Medical Economics* **2016**, *19*, 135-147.
11. Statistics Canada. Average usual hours and wages by selected characteristics, monthly, unadjusted for seasonality (Table: 14-10-0320-02). Available online: https://www150.statcan.gc.ca/t1/tbl1/en/tv.action?pid=1410032002 (accessed on 1 October 2020).
12. Cancer Care Ontario. AZCTVENE Regimen. Available online: https://www.cancercareontario.ca/en/drugformulary/regimens/monograph/66881 (accessed on 25 November 2020).
13. Cancer Care Ontario. CYTA(LD) Regimen. Available online: https://www.cancercareontario.ca/en/drugformulary/regimens/monograph/51351 (accessed on 1 November 2020).
14. Ontario Ministry of Health and Long Term Care. Ontario Care Costing Analysis Tool. Available online: https://hsimi.ca/occp/occpreports/(accessed on 1 October 2020).
15. American Cancer Society. Stem Cell Transplant for Acute Myeloid Leukemia (AML). Available online: https://www.cancer.org/cancer/acute-myeloid-leukemia/treating/bone-marrow-stem-cell-transplant.html (accessed on 1 October 2020).
16. Interprovincial Health Insurance Agreements Coordinating Committee (IHIACC). Interprovincial Billing Rates for Designated High Cost Transplants Effective for Discharges on or after April 1, 2020. Available online: http://www.health.gov.on.ca/en/pro/programs/ohip/bulletins/na_86/high_cost.pdf (accessed on 1 October 2020).
17. Canadian Institute for Health Information. Patient Cost Estimator. Available online: https://www.cihi.ca/en/patient-cost-estimator (accessed on 1 October 2020).
18. Canadian Institute for Health Information. Care in Canadian ICUs. Available online: https://secure.cihi.ca/free_products/ICU_Report_EN.pdf (accessed on 1 October 2020).
19. Lagerquist O; Poseluzny D; Werstiuk G; Slomp J; Maier M; S, N.; G, C. The cost of transfusing a unit of red blood cells: a costing model for Canadian hospital use. *International Society of Blood Transfusion* **2017**, *12*, 375-380.
20. Canadian Cancer Society. Follow-up after treatment for acute myelogenous leukemia. Available online: https://www.cancer.ca/en/cancer-information/cancer-type/leukemia-acute-myelogenous-aml/treatment/follow-up/?region=on (accessed on 1 October 2020).
21. Ministry of Health and Long Term Care of Ontario. Schedule of Benefits of Laboratory Services (Effective July 1, 2020). Available online: http://www.health.gov.on.ca/en/pro/programs/ohip/sob/lab/lab_mn2020.pdf (accessed on 1 October 2020).
22. Oliveira C; Pataky R; Bremner K.E; Rangrej J; Chan K.K.W; Cheung W.Y; Hoch J.S; S, P.; M.D, K. Phase-specific and lifetime costs of cancer care in Ontario, Canada *BMC Cancer* **2016**, *16*, 1-12.
23. Statistics Canada. Labour force characteristics by age group, monthly, seasonally adjusted (Table: 14-10-0287-02). Available online: https://www150.statcan.gc.ca/t1/tbl1/en/cv.action?pid=1410028702 (accessed on 1 October 2020).
